# CTCF Represses CIB2 to Balance Proliferation and Differentiation of Goat Myogenic Satellite Cells via Integrin α7β1–PI3K/AKT Axis

**DOI:** 10.3390/cells14151199

**Published:** 2025-08-05

**Authors:** Changliang Gong, Huihui Song, Zhuohang Hao, Zhengyi Zhang, Nanjian Luo, Xiaochuan Chen

**Affiliations:** 1 Chongqing Key Laboratory of Herbivore Science, College of Animal Science and Technology, Southwest University, Chongqing 400715, China; m15083461065@163.com (C.G.); 18893492303@163.com (H.S.); hzh000602@163.com (Z.H.); 2 School of Preclinical Medicine, Zunyi Medical University, Zunyi 563000, China; zzy071011@sina.com

**Keywords:** *CIB2*, *CTCF*, goat, proliferation, breeding strategies

## Abstract

Skeletal muscle development is a critical economic trait in livestock, governed by myogenic satellite cell regulation. Integrins mediate mechanical anchorage to the ECM and enable ECM–intracellular signaling. *CIB2*, as an EF-hand-domain protein involved in mechanotransduction, shows significant developmental regulation in goat muscle. Although the role of *CIB2* in skeletal muscle growth is poorly characterized, we observed pronounced developmental upregulation of *IB2* in postnatal goat muscle. *CIB2* expression increased >20-fold by postnatal day 90 (P90) compared to P1, sustaining elevation through P180 (*p* < 0.05). Functional investigations indicated that siRNA-mediated knockdown of *CIB2* could inhibit myoblast proliferation by inducing S-phase arrest (*p* < 0.05) and downregulating the expression of *CDK4*/*Cyclin D*/*E*. Simultaneously, *CIB2* interference treatment was found to decrease the proliferative activity of goat myogenic satellite cells, yet it significantly promoted differentiation by upregulating the expression of *MyoD*/*MyoG*/*MyHC* (*p* < 0.01). Mechanistically, *CTCF* was identified as a transcriptional repressor binding to an intragenic region of the *CIB2* gene locus (ChIP enrichment: 2.3-fold, *p* < 0.05). Knockdown of *CTCF* induced upregulation of *CIB2* (*p* < 0.05). RNA-seq analysis established *CIB2* as a calcium signaling hub: its interference activated IL-17/TNF and complement cascades, while overexpression suppressed focal adhesion/ECM–receptor interactions and enriched neuroendocrine pathways. Collectively, this study identifies the CTCF-CIB2–integrin α7β1–PI3K/AKT axis as a novel molecular mechanism that regulates the balance of myogenic fate in goats. These findings offer promising targets for genomic selection and precision breeding strategies aimed at enhancing muscle productivity in ruminants.

## 1. Introduction

Goats represent significant livestock genetic resources in Southwest China, exhibiting advantages such as tolerance for coarse feed and robust disease resistance. However, their small size, slow growth rate, and low slaughter rate constrain the realization of their genetic potential [1,2]. Skeletal muscle, a core economic trait, relies on the proliferation and differentiation efficiency of MuSCs for its developmental quality. These multipotent progenitor cells dynamically regulate myofiber formation through quiescence–activation transitions [3]. This process is precisely controlled by the interaction between the ECM and integrins. Muscle fibers are embedded within a three-dimensional ECM network composed of components such as collagen and laminin. Integrins, functioning as transmembrane receptors, provide structural anchorage for muscle cells and activate downstream signaling pathways via specific binding to ECM components [4,5]. These interactions play critical roles in maintaining skeletal muscle development and regeneration.

Within the skeletal muscle system, the integrin α7β1 subtype holds particular importance. Deficiency in this integrin can lead to muscular dystrophy and congenital muscular atrophy [6]. Integrins coordinate muscle cell behavior through a bidirectional regulatory mechanism: externally, their extracellular domains bind to the ECM, activating the FAK/PI3K/MAPK signaling axis to drive processes such as adhesion, migration, and differentiation; internally, intracellular signals enhance integrin affinity for the ECM, establishing a self-reinforcing feedback loop [5,7]. *CIB2*, a member of the calcium- and integrin-binding protein family, features four EF-hand domains and transmembrane helices [8,9]. In the cardiovascular system, *CIB2* cooperates with integrin β1D to modulate RyR2 channel calcium homeostasis in cardiomyocytes; abnormal *CIB2* expression exacerbates arrhythmia [10]. In retinal pigment epithelial cells, *CIB2* deletion perturbs integrin interactions, leading to abnormal mTORC1 signaling pathway activation and autophagy defects [11]. This mechanism aligns with integrin α5β3-mediated photoreceptor cell structural damage observed in Usher syndrome retinopathy [12]. While these studies delineate CIB2–integrin functional partnerships in cardiovascular and retinal systems, a systematic investigation into their cooperative roles during mammalian skeletal muscle development remains elusive.

The human *CIB2* gene is located in the q24 region of chromosome 15 (15q24) [13]. Patients with interstitial deletions in this region exhibit clinical symptoms such as hypotonia and significant developmental delay [14]. Studies on local Polish pig breeds indicate that *CIB2* expression is significantly upregulated in the *longissimus dorsi* of high-meat-yielding Polish Large White fattening pigs [15]. However, research on *CIB2* function in skeletal muscle cells remains limited, with only a few studies suggesting an association between *CIB2* expression levels and skeletal muscle growth [16]. In this study, we systematically investigated the regulatory role of *CIB2* in goat myogenic satellite cell proliferation and differentiation. Using *CIB2* interference and overexpression techniques, we performed RNA-seq analysis on goat myogenic satellite cells to elucidate the intrinsic connections and specific regulatory mechanisms underlying their cell fate determination. Collectively, this study defines the regulatory hierarchy of *CIB2* in myogenic satellite cell fate determination, providing a mechanistic framework for its role in skeletal muscle development.

## 2. Materials and Methods

### 2.1. Animal and Sample Collection

All experimental procedures were approved by the Institutional Animal Care and Use Committee (IACUC) of Southwest University (Approval No. GB14925-2010). Tissue samples were obtained from a native Chinese goat breed (Dazu Black Goat, *Capra hircus*). Kids purchased from Tengda Ranch (Chongqing, China) were humanely euthanized at 1–2 days of age via intravenous injection of sodium pentobarbital through the marginal ear vein. Organs and tissues were immediately weighed, flash-frozen in liquid nitrogen, and stored at −80 °C for subsequent analysis. The longissimus dorsi muscle was aseptically collected for primary cell isolation.

### 2.2. Isolation, Purification, and Characterization of MuSCs

Skeletal muscle tissue was obtained from the longissimus dorsi of 1–2-day-old Dazu black goats [17]. Specifically, tissue fragments were rinsed in ice-cold phosphate-buffered saline supplemented with 4% penicillin–streptomycin solution (PS, C0222, Beyotime Biotechnology, Shanghai, China), mechanically minced into approximately 1 mm^3^ pieces, and then subjected to sequential enzymatic digestion. Initially, the tissue was digested with 0.2% collagenase type I (C8140, Solarbio, Beijing, China) for 60 min at 37 °C with continuous agitation, followed by digestion with 0.25% trypsin (C25200072, Gibco, Grand Island, NY, USA) for 10 min in a water bath at 37 °C. The digestion process was terminated by the addition of complete culture medium (DMEM/F-12 supplemented with 10% fetal bovine serum and 1% PS, C11330500BT, Gibco, Grand Island, NY, USA). The resulting cell suspension was sequentially filtered through 200 μm and 38 μm meshes, centrifuged, resuspended in fresh medium, and seeded into T25 culture flasks. Cells were cultured at 37 °C under a humidified atmosphere containing 5% CO_2_, with medium changes every 48 h. After 24 h of primary culture, non-adherent cells were carefully transferred to new T25 flasks to enhance cell yield. Briefly, cells were purified by Percoll gradient centrifugation and identified by Pax7 (20570-1-AP, Chromotek, Shanghai, China) immunofluorescence staining.

### 2.3. Cell Culture

We seeded MuSCs in growth medium (GM: DMEM/F-12 with 10% FBS and 1% PS) and maintained them under a humidified atmosphere of 5% CO_2_. When the cells reached 80–90% confluence, they were harvested using 0.25% trypsin, resuspended, and reseeded into 6-well plates at a density of 1 × 10^4^ to 1 × 10^5^ cells per well. To induce myogenic differentiation, the growth medium was replaced with differentiation medium (DM: DMEM (10099-141, Gibco, Frederick, MD, USA) supplemented with 2% horse serum (Gibco) and 2% PS) once the cells reached 80–90% confluence.

C2C12 mouse myoblasts were obtained from Shanghai Anwei Biotechnology (Shanghai, China). The cells were cultured in proliferation medium (DMEM supplemented with 10% FBS and 1% PS) at 37 °C in a humidified atmosphere containing 5% CO_2_, with the medium replaced every 48 h.

### 2.4. Overexpression Plasmid Construction, siRNA Preparation, and Oligonucleotide Synthesis

Two *CIB2*-specific siRNA and shRNA sequences were designed for mouse C2C12 cells and goat MuSCs, respectively, based on NCBI reference sequences (mouse: NM_019686.6; goat: XM_005695085.3) following standard siRNA design principles. Similarly, two siRNA sequences targeting the *CTCF* gene (mouse: NM_001358924.2) were designed using identical criteria. All siRNA sequences and validation primers are detailed in Supplementary Table S1.

The full-length coding sequence (CDS) of the goat *CIB2* gene (XM_005695085.3) was PCR-amplified using its NCBI reference sequence as template. The purified product was digested with restriction enzymes and ligated into the pcDNA3.1(+) eukaryotic expression vector to generate the pcDNA3.1-*CIB2* recombinant plasmid. The empty pcDNA3.1(+) vector served as the negative control (NC). Vector construction and Sanger sequencing verification were performed by Shanghai Sangon Biotech.

### 2.5. Flow Cytometry

Cells were maintained in standard growth medium and passaged at 70% confluence. Transfection was performed with Lipofectamine 2000 (11668019, Carlsbad, CA, USA) at 60% confluence. Forty-eight hours post-transfection, cell cycle distribution was analyzed using the Cell Cycle and Apoptosis Analysis Kit (C1052, Beyotime Biotechnology, Shanghai, China). Cells were fixed in 70% ethanol, RNase A-treated, and stained with propidium iodide (PI). DNA content was quantified on a FACSCalibur flow cytometer (342975, BD Biosciences, San Jose, CA, USA) using 488 nm excitation.

For apoptosis detection, cells were stained with FITC-Annexin V/PI (C1062, Beyotime Biotechnology, Shanghai, China), incubated for 15 min in the dark, and immediately analyzed on the FACSCalibur. A minimum of 20,000 events per sample were recorded and analyzed using FlowJo v10.8 software (BD Biosciences, San Jose, CA, USA).

### 2.6. CCK-8 Cell Viability and EdU Proliferation Assays

For viability assessment, cells were seeded in 96-well plates (1000 cells/well), cultured overnight, and transfected with Lipofectamine 2000 (11668019, Invitrogen, Carlsbad, CA, USA). Metabolic activity was quantified at 24, 48, and 72 h post-transfection using Cell Counting Kit-8 (C0037, Beyotime Biotechnology, Shanghai, China). Cell Counting Kit-8 (CCK-8) reagent (10 μL/well) was added, followed by 2 h incubation at 37 °C. Absorbance (450 nm) was measured using a Varioskan™ LUX microplate reader (VL0000D0, Thermo Fisher Scientific, San Diego, CA, USA).

For proliferation analysis, cells seeded in 12-well plates were transfected after 12 h incubation using Lipofectamine 2000. EdU incorporation was detected using the BeyoClick™ EdU-488 Kit (C0071S, Beyotime Biotechnology, Shanghai, China) according to manufacturer specifications. EdU-positive nuclei were visualized under an Axio Observer inverted fluorescence microscope (ZEISS, Oberkochen, Germany) and quantified against total nuclei using ImageJ v1.53 (National Institutes of Health, Bethesda, MD, USA).

### 2.7. Immunofluorescence

Isolated muscle tissues were embedded in OCT™ compound (Sakura Finetek, Torrance, CA, USA) and cryosectioned. Sections were fixed in 4% paraformaldehyde (PFA) for 15 min at room temperature (RT), and then incubated overnight at 4 °C with anti-CIB2 primary antibody (AF0349, Affinity Biosciences, Cincinnati, OH, USA). After three 5 min PBS washes, sections were incubated for 1 h at RT with FITC-conjugated anti-rabbit IgG secondary antibody (111-095-003, Jackson Immuno Research, West Grove, PA, USA). Following final PBS washes, sections were mounted in Fluoroshield with DAPI (F6057, Sigma-Aldrich, Darmstadt, Germany) and imaged using an Axio Imager Z2 epifluorescence microscope (ZEISS, Tokyo, Japan). GFP-positive cells were quantified across five random fields per sample using ImageJ v1.53 (National Institutes of Health, Bethesda, MD, USA).

### 2.8. RNA Sequencing

RNA sequencing was performed by IGENEBOOK Biotechnology (Wuhan, China). Total RNA was extracted from *CIB2* knockdown, overexpression, and control groups using the RNAprep Pure Kit (DP432, TIANGEN, Beijing, China). RNA integrity was verified (RIN > 8.0) via the Qsep1 Bio-Fragment Analyzer (F0130, BiOptic Inc., Taiwan, China). Libraries were prepared from 1 μg high-quality RNA using the MGIEasy mRNA Library Prep Kit (1000004155, MGI Tech, Shenzhen, China) and sequenced (150 bp paired-end) on the DNBSEQ-G400 platform (MGI). Raw sequencing data were processed to remove low-quality reads using cutadapt (v1.11), followed by alignment to the goat reference genome using Hisat2 (v2.1.0). Transcript expression levels were quantified and normalized based on fragments per kilobase of transcript per million mapped reads (FPKM). Differentially expressed genes were identified using the edgeR algorithm, with the false discovery rate (FDR) < 0.05 and |log_2_(fold change)| ≥ 1.0 set as the threshold for significantly differential expression [18,19]. GO enrichment analysis of the differentially expressed genes (DEGs) was implemented by the GOseq R packages (V1.60.0) based on Wallenius’ non-central hyper-geometric distribution [20]. GO and Kyoto Encyclopedia of Genes and Genomes (KEGG) terms with corrected *p* < 0.05 were defined as significantly enriched by commonly expressed genes (CEGs) and DEGs [20].

### 2.9. Chromatin Immunoprecipitation–Quantitative PCR (ChIP–qPCR)

ChIP-qPCR was performed to validate *CTCF*–*CIB2* regulatory interactions. Briefly, crosslinked cellular lysates were sonicated to fragment chromatin into 200–500 bp fragments, followed by immunoprecipitation using an anti-*CTCF* antibody (2899S, Cell Signaling Technology, Danvers, MA, China). Input controls, representing 10% of the total sample volume, were reserved for normalization. Immunoprecipitated DNA was purified using the Universal DNA Purification Kit (DP214, TIANGEN) and subjected to quantitative PCR analysis.

Bioinformatic predictions were conducted through integrated database analyses. Potential transcription factors binding to *CIB2* were predicted using hTFtarget (https://guolab.wchscu.cn/hTFtarget/#!/, 8 October 2023). Binding peak intensities between *CIB2* and candidate transcription factors were assessed via Cistrome DB (http://cistrome.org/db/#/, 20 October 2023). Putative binding regions were further mapped using the UCSC Genome Browser (https://genome.ucsc.edu/, 8 November 2023). The primer sequences utilized for ChIP-qPCR amplification are provided in Supplementary Table S2.

### 2.10. Isolation of Total RNA and Performance of RT–qPCR

Total RNA was isolated using Trizol reagent (15596026CN, Invitrogen, Carlsbad, CA, USA) according to the manufacturer’s instructions. The concentration and purity of RNA were determined using a Nanodrop One spectrophotometer (Thermo Fisher Scientific, Waltham, MA, USA). mRNA was reverse-transcribed into cDNA using the PrimeScript RT Kit and gDNA Eraser (RR047A, Tiangen Biotech Co., Shanghai, China) following the manufacturer’s protocol. The resulting cDNA was stored at −20 °C for future use. The relative expression levels of mRNA were determined using TB Green Premix Ex Taq II (RR820A, Takara, Tokyo, Japan) and the CFX96 Touch Real-Time PCR Detection System (Bio-Rad, Hercules, CA, USA). The thermal cycling conditions for RT-qPCR were as follows: 95 °C for 30 s; 95 °C for 5 s; and 60 °C for 30 s, for a total of 40 cycles. The relative level of mRNA expression was calculated by using the 2^−△△CT^ method after normalization with *GAPDH* as a housekeeping gene. The primers used in this study are listed in Supplementary Table S3.

### 2.11. Statistical Analysis

The relative expression data of the *CIB2* gene in goat muscle tissue at pre- and postnatal stages were derived from the Ruminant Genome Database (http://animal.omics.pro/code/index.php/RGD/searchByGeneGBI, 8 October 2023). This database integrates publicly accessible RNA-seq datasets across developmental stages.

All data are shown as the means ± SEMs. Student’s *t* test (two groups) or one-way ANOVA followed by Dunnett’s post hoc test was employed for comparisons between the groups using GraphPad Prism Software Version 5.9. Values of *p* < 0.05 were considered statistically significant, and *p* < 0.01 was considered significantly different.

## 3. Results

### 3.1. Developmental Function and Evolutionary Conservation of Caprine CIB2

**Figure 1:**
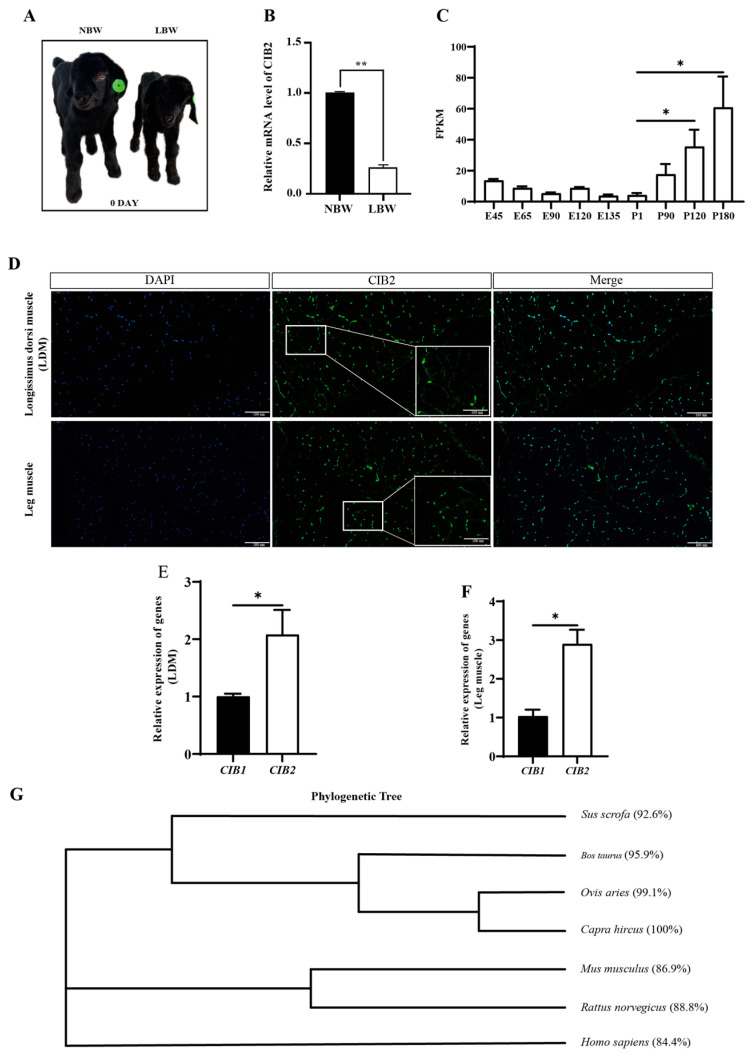
Developmental function and evolutionary conservation of caprine *CIB2*. (**A**) Comparison of normal-birth-weight (NBW) and low-birth-weight (LBW) lambs (1 day). (**B**) The mRNA levels of *CIB2* in the skeletal muscle (*n* = 3). (**C**) Developmental changes in CIB2 mRNA expression (FPKM) in goat skeletal muscle. (**D**) The expression level of *CIB2* in longissimus dorsi muscle and leg muscle was determined by tissue immunofluorescence (scale bar, 50 μm, 30 days). (**E**) The different mRNA expression levels of *CIB1* and *CIB2* in goat longissimus dorsi muscle. (**F**) The different mRNA expression levels of *CIB1* and *CIB2* in goat leg muscle. (**G**) Homology analysis of *CIB2* gene in different species. The data are shown as the means ± SEMs. * *p* < 0.05; ** *p* < 0.01.

Our previous studies demonstrated a significant reduction (*p* < 0.05) in *CIB2* expression in the skeletal muscle of low-birth-weight (LBW) Dazu black goats compared to their normal-birth-weight counterparts (Figure 1A,B) [21]. Analysis of publicly available RNA-seq datasets across postnatal developmental stages revealed a progressive increase in *CIB2* expression, with levels rising approximately 20-fold at postnatal day 120 (P120) and 26-fold at P180 relative to P1 muscle (*p* < 0.05; Figure 1C). Immunofluorescence staining of the longissimus dorsi and hindlimb muscles confirmed distinct subcellular localization of *CIB2*, characterized by membrane-associated signals and nuclear puncta (Figure 1D).

Given the structural homology among CIB family members, we systematically compared their expression profiles in skeletal muscle. RT-qPCR analysis identified *CIB2* as the functionally dominant isoform, with expression levels in the longissimus dorsi and hindlimb muscles significantly higher than those of *CIB1* (*p* < 0.05; Figure 1E,F). *CIB3* and *CIB4* transcripts were undetectable, suggesting that *CIB2* serves as the core functional subtype in caprine skeletal muscle.

Cross-species homology analysis using the goat *CIB2* reference sequence (GenBank: XM_005695085.3) revealed high sequence conservation among artiodactyls, with 99.1% identity to *Ovis aries*, 95.9% to *Bos taurus*, and 92.6% to *Sus scrofa*. Notably, significant conservation was also observed beyond the order Artiodactyla, showing 86.9% homology with *Mus musculus* and 84.4% with *Homo sapiens* (Figure 1G). This pattern of evolutionary conservation implies the presence of conserved regulatory mechanisms across mammalian species.

### 3.2. CIB2 Promotes Myoblast Proliferation Through Cell Cycle Regulation

**Figure 2:**
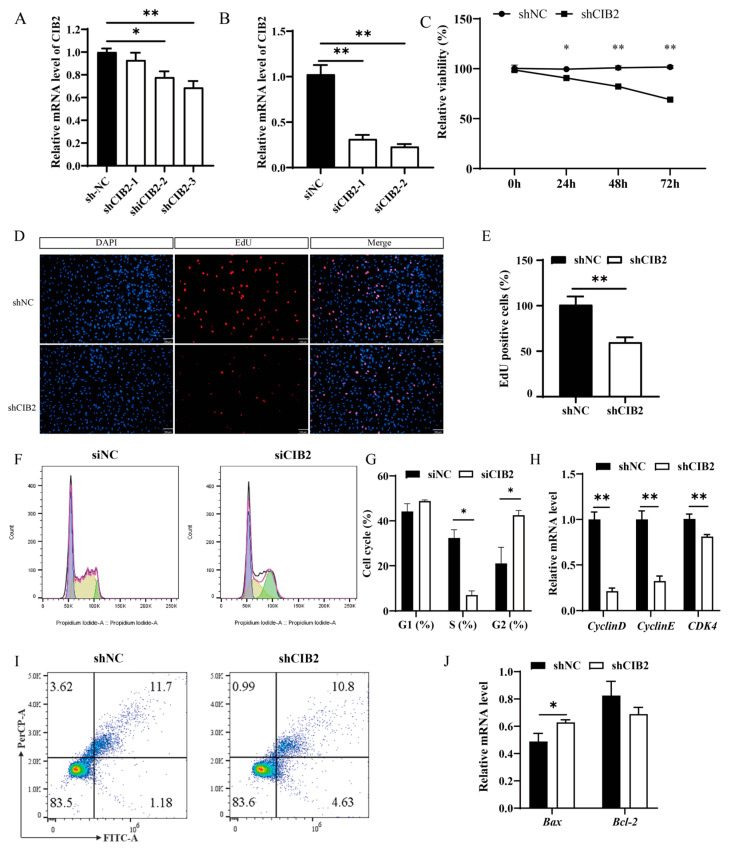
Effect of *CIB2* on the proliferation and apoptosis of C2C12 myoblast cells. (**A**) Assessment of the interference efficiency of the *CIB2* mRNA using shRNA (*n* = 3). (**B**) Assessment of the interference efficiency of the *CIB2* mRNA using siRNA (*n* = 3). (**C**) CCK8 assay for cell viability of shCIB2-transfected C2C12 myoblast cells. (**D**,**E**) EdU (scale bar, 100 μm) was used to measure the proliferative capacity. (**F**) Representative flow cytometry histograms showing cell cycle distribution. (**G**) Quantitative analysis of cells in G1, S, and G2 phases based on panel F (*n* = 3). (**H**) mRNA expression levels of cell cycle marker genes (*n* = 3). (**I**) Flow cytometry analysis of apoptosis rates (*n* = 3). (**J**) mRNA expression levels of apoptosis marker genes. The data are shown as the means ± SEMs. * *p* < 0.05; ** *p* < 0.01.

Given the dynamic expression pattern of *CIB2* during muscle development, we designed an interference fragment targeting the *CIB2* gene. The results indicated that the highest interference efficiency achieved by groups using shRNA and siRNA was 25% and 70%, respectively (*p* < 0.05; Figure 2A,B). *CIB2* knockdown significantly reduced cell viability at both 24 h and 48 h post-transfection (*p* < 0.01; Figure 2C). EdU incorporation was reduced by 40% in the knockdown groups compared to controls (*p* < 0.05; Figure 2D,E).

Flow cytometric analysis of the cell cycle revealed that *CIB2* knockdown decreased S-phase population and increased G2/M-phase distribution (*p* < 0.05; Figure 2F,G). Consistently, RT-qPCR analysis showed decreased mRNA expression of the cell cycle regulators *Cyclin E* and *CDK4* (*p* < 0.05), while *Cyclin D* expression remained unchanged (Figure 2H).

Although *Bax* expression increased 1.5-fold in knockdown cells (*p* < 0.05; Figure 2J), flow cytometry revealed no significant difference in apoptosis rates versus controls (*p* > 0.05; Figure 2I). These data suggest that *CIB2* regulates myoblast proliferation primarily through cell cycle modulation rather than apoptotic pathways.

### 3.3. CIB2 Suppresses Proliferation While Enhancing Differentiation of Caprine MuSCs

#### 3.3.1. Isolation and Validation of Caprine MuSCs

**Figure 3:**
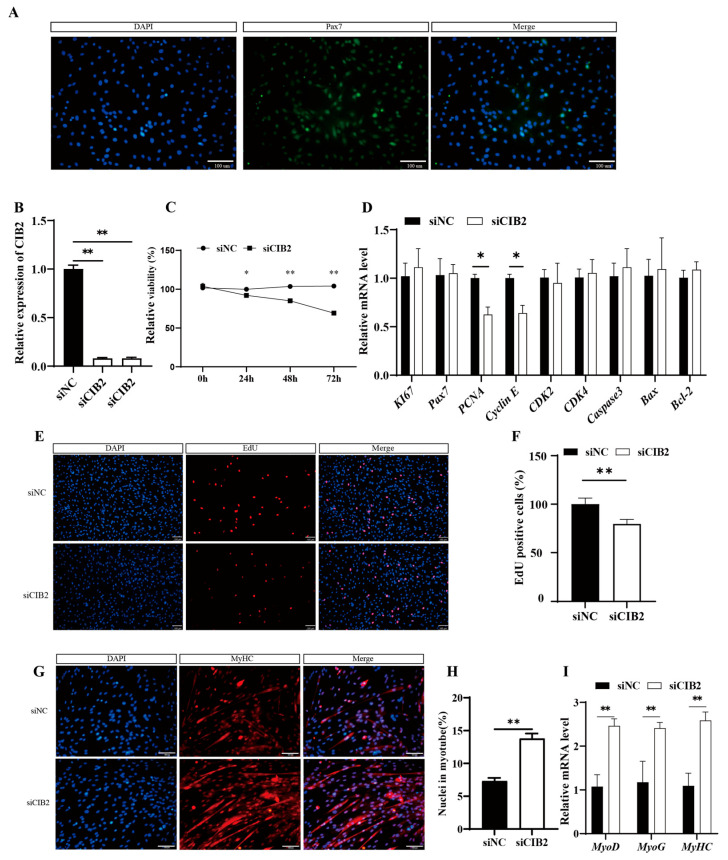
Effect of *CIB2* on the proliferation and differentiation of goat MuSCs. (**A**) Control of purity of goat myogenic satellite cell preparation. (**B**) Relative mRNA expression of *CIB2* after interference of *CIB2* (*n* = 3). (**C**) CCK8 assay for cell viability of siCIB2-transfected goat MuSCs. (**D**) RT-qPCR was used to detect the effect of *CIB2* interference on the expression of cell proliferation- and apoptosis-related genes (*n* = 3). (**E**,**F**) EdU was used to measure the proliferative capacity (scale bar, 100 μm). (**G**) Immunofluorescence was used to measure differentiation marker *MyHC* protein expression (scale bar, 100 μm). (**H**) Myotube nuclear occupancy rate at differentiation day 5 (*n* = 3). (**I**) RT-qPCR was used to detect the effect of *CIB2* interference on the expression of cell differentiation-related genes (*n* = 3). The data are shown as the means ± SEMs. * *p* < 0.05; ** *p* < 0.01.

During the isolation of muscle stem cells, fibroblast contamination may occur. To evaluate the purity of the isolated goat muscle-derived satellite cells, immunofluorescence staining was performed to detect the expression of *Pax7*, a specific marker protein for muscle satellite cells. The results obtained using *Pax7*-specific immunofluorescence staining demonstrated high *Pax7* protein expression in the isolated cells (Figure 3A), consistent with the characteristic features of satellite cells, thereby confirming the successful acquisition of high-purity goat MuSCs.

#### 3.3.2. Interference with the CIB2 Gene Inhibits the Proliferation of Goat MuSCs

Two siRNAs targeting *CIB2* were transfected into MuSCs. siRNA#2 achieved the highest knockdown efficiency, with a 70% reduction in mRNA levels (*p* < 0.01; Figure 3B). Depletion of *CIB2* significantly reduced cell viability at 24 h, 48 h, and 72 h post-transfection (*p* < 0.01; Figure 3C).

RT-qPCR analysis revealed a 1.6-fold decrease in *PCNA* mRNA and a 1.5-fold decrease in *Cyclin E* mRNA (*p* < 0.05; Figure 3D). EdU incorporation assays demonstrated a 10% reduction in EdU-positive cells in the knockdown groups (*p* < 0.05; Figure 3E,F).

#### 3.3.3. CIB2 Knockdown Promotes Myogenic Differentiation

At 48 h post-knockdown, immunofluorescence analysis shows a 1.7-fold increase in the differentiation index compared to controls (*p* < 0.01; Figure 3G,H). Furthermore, qPCR analysis supported that the mRNA expression levels of key muscle differentiation regulators, *MyoD*, *MyoG*, and *MyHC*, were markedly upregulated by 2.1-fold, 1.8-fold, and 2.3-fold, respectively (*p* < 0.01, Figure 3I). Collectively, these results demonstrate that inhibition of *CIB2* gene expression promotes myogenesis in goats.

### 3.4. CTCF Negatively Regulates CIB2 mRNA Expression

#### 3.4.1. Multi-Omics Analysis Identifies CTCF as a Putative CIB2 Regulator

**Figure 4:**
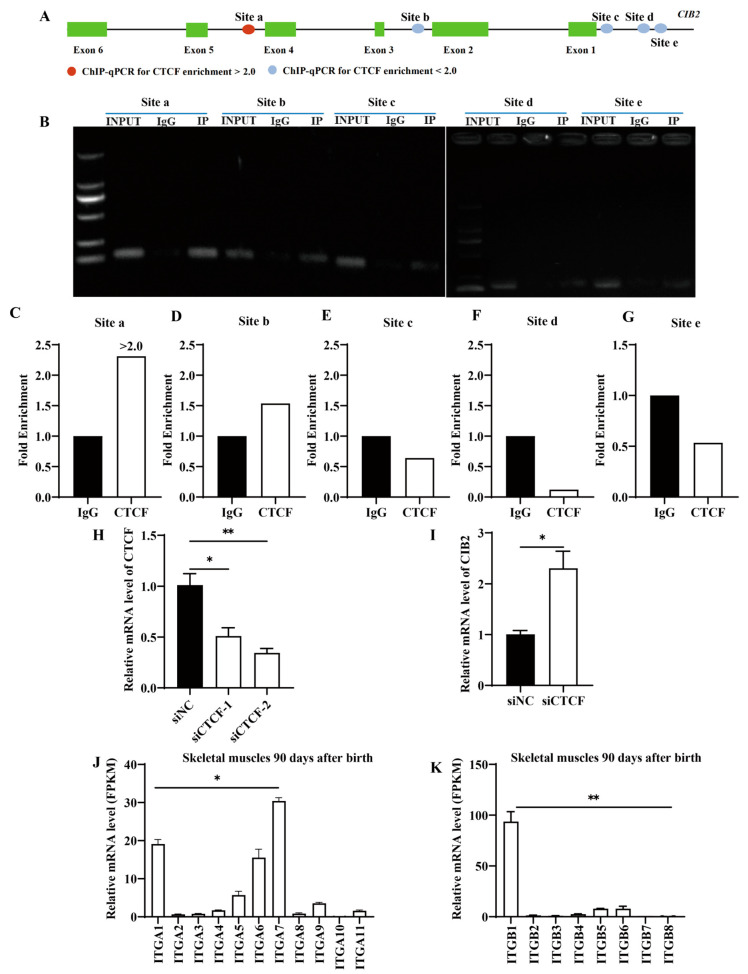
*CTCF* regulation of *CIB2* expression. (**A**) The potential binding sites of *CTCF* on the *CIB2* gene. (**B**) Agarose gel electrophoresis of *CIB2* gene binding to *CTCF*. (**C**–**G**) ChIP-qPCR verified the relative enrichment of *CTCF* at different binding sites of *CIB2* gene. (**H**) Relative mRNA expression of *CTCF* after interference of *CTCF* (*n* = 3). (**I**) Relative expression of *CIB2* after interference of *CTCF* (*n* = 3). (**J**) mRNA expression of integrin α-subtypes in 90-day skeletal muscle (*n* = 3, RNA-seq). (**K**) mRNA expression of integrin β-subtypes in 90-day skeletal muscle (*n* = 3, RNA-seq). The data are shown as the means ± SEMs. * *p* < 0.05; ** *p* < 0.01.

Bioinformatic screening of 150 transcription factors revealed *CTCF* as the top candidate binding to *CIB2* based on peak intensity (Table 1). Five putative *CTCF*-binding sites were predicted within the *CIB2* locus (Figure 4A).

#### 3.4.2. ChIP-qPCR Validation of CTCF-Binding Sites

We designed primers flanking five candidate regions (a–e) in the *caprine CIB2* locus. ChIP-qPCR indicated significant *CTCF* enrichment specifically at site a (2.3-fold vs. IgG control; *p* < 0.05), exceeding our significance threshold (fold enrichment > 2.0) (Figure 4B,C). No enrichment was detected at sites b–e (Figure 4D–G).

#### 3.4.3. CTCF Represses CIB2 Transcription

siRNA-mediated *CTCF* knockdown in caprine MuSCs reduced *CTCF* mRNA by 60% versus the scramble control (*p* < 0.01; Figure 4H). This depletion significantly upregulated *CIB2* expression 2.0-fold (*p* < 0.05; Figure 4I), demonstrating direct transcriptional repression of *CIB2* by *CTCF*.

### 3.5. Analysis of Interaction Between CIB2 and Integrins

Transcriptomic analysis of public datasets revealed significant postnatal upregulation of *CIB2* in caprine skeletal muscle, suggesting its functional importance in myogenesis. Parallel screening of integrin subunits identified *ITGA7* and *ITGB1* as the most abundantly expressed subtypes in developing muscle (*p* < 0.05; Figure 4J,K).

Structural predictions using AlphaFold 3 further validated direct interaction interfaces between *CIB2* and integrin α7β1, with specific pairwise interaction scores as follows: *CIB2*-*ITGA7* (pTM = 0.70), *CIB2*-*ITGB1* (pTM = 0.63), and the ternary complex (pTM = 0.56) (Figure S1). Collectively, these expression patterns and structural data provide a robust mechanistic foundation for CIB2’s regulation of the integrin signaling pathway during skeletal muscle development.

### 3.6. Altered CIB2 Expression Disrupts Critical Regulatory Pathways in Caprine MuSCs

#### 3.6.1. Differential Gene Expression Profiling

**Figure 5:**
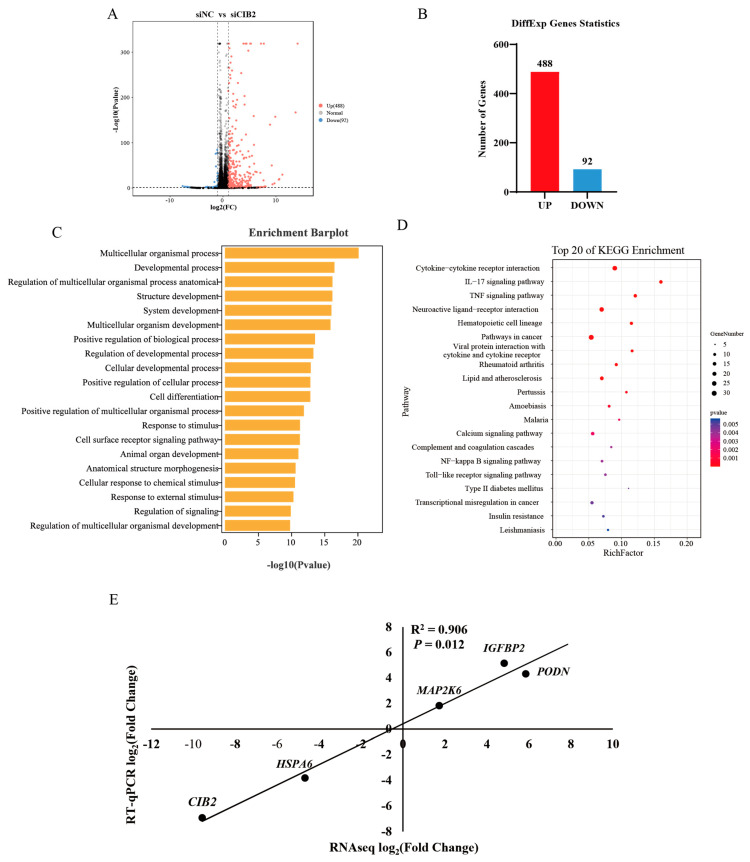
Transcriptome sequencing analysis of goat myogenic satellite cells after interference with *CIB2* (*n* = 3). (**A**) Volcano map of differentially expressed genes after interference with *CIB2*. (**B**) The number of differentially expressed genes after interference with *CIB2*. (**C**) GO biological process (BP) enrichment analysis following *CIB2* interference highlighted the top 15 terms with the lowest *p*-values. (**D**) KEGG enrichment analysis of differentially expressed genes after interference with *CIB2*. (**E**) Validation of RNA-seq data in the *CIB2* interference group by RT-qPCR. The slope of best fit after Pearson correlation was 0.95, with a 95% confidence interval of 0.425 to 1.199.

To investigate CIB2’s functional role, we performed *CIB2* knockdown using siRNA in caprine MuSCs and overexpression via plasmid transfection, followed by RNA extraction 48 h post-treatment. RNA-seq analysis revealed 580 DEGs following *CIB2* knockdown (488 upregulated, 92 downregulated), including *COL13A1*, *CDHR3*, and *MYOG* (Figure 5A,B). Statistics of sequencing data output, including the quality score and size of trimmed sequences, are presented in Supplementary Table S3.

#### 3.6.2. GO Enrichment Analysis

Upon *CIB2* interference, GO enrichment analysis of differentially expressed genes revealed that upregulated genes predominantly influenced core pathways: the developmental regulation pathway (177 upregulated genes) activated muscle differentiation and tissue development functions; the signal transduction pathway (156 upregulated genes), including *Wnt* and *TGF-β*, was significantly enhanced; and the cellular process pathway (308 upregulated genes) exhibited metabolic reprogramming and intensified cytoskeletal reorganization (Figure 5C). These findings suggest that *CIB2* bidirectionally maintains cellular homeostasis by modulating developmental differentiation and signal transduction networks through inhibition or activation.

#### 3.6.3. KEGG Enrichment Analysis

*CIB2* bidirectionally regulates immune homeostasis and tissue structure and function through the calcium signaling hub. Specifically, *CIB2* interference significantly activates the *IL-17*/*TNF* signaling pathway, complement and coagulation cascades, and cytokine networks, thereby driving inflammatory responses and disrupting immune homeostasis via the calcium signaling–immune axis (Figure 5D). In contrast, *CIB2* overexpression enriches pathways related to focal adhesions, ECM–receptor interactions, glutamatergic/GABAergic synapses (neural conduction), and insulin/thyroid hormone pathways (endocrine regulation), thus coordinating cell adhesion and neuroendocrine homeostasis through the calcium signaling–structural and functional axis (Figure S2). Notably, the complement pathway is consistently enriched in both experimental conditions, suggesting that *CIB2* plays a regulatory role in the complement system.

#### 3.6.4. Transcriptomic Validation

To ensure the reliability of the sequencing data, RT-qPCR validation was performed on nine randomly selected DEGs. The correlation coefficient (R) for the fold changes between the two methods was 0.906, demonstrating that the differentially expressed genes identified by RNA-seq exhibited highly consistent expression patterns when validated using RT-qPCR (*p* < 0.05; Figure 5E).

## 4. Discussion

Birth weight serves as a critical economic trait for evaluating early lamb development, directly influencing caprine growth performance (e.g., meat yield) and providing a phenotypic basis for genetic selection [22,23,24]. Muscle development—a dynamically regulated process—centrally governs livestock meat production [25,26]. Our integrated analysis establishes *CIB2* as a pivotal regulator of goat myogenesis, coordinating satellite cell proliferation and differentiation through discrete molecular pathways. Functional studies show that *CIB2* depletion induces S-phase arrest via *CDK4*/*Cyclin D*/*E* suppression, impairing myoblast expansion, while paradoxically accelerating differentiation through *MyoD*/*MyoG*/*MyHC* activation. Mechanistically, ChIP-qPCR confirms CTCF-mediated transcriptional repression of *CIB2*, revealing an epigenetic control layer. Transcriptomic profiling further demonstrates CIB2’s role as a signaling hub, modulating PI3K/AKT and ECM–integrin pathways. These data collectively position *CIB2* within a conserved regulatory axis (CTCF-CIB2–integrin α7β1) that balances muscle development—a mechanism potentially exploitable for enhancing livestock productivity.

The core process of skeletal muscle development hinges on the precise regulation of myoblast proliferation, differentiation, and their ultimate fusion into functional multinucleated myofibers [27,28,29,30]. While prior studies have shown that *CIB2* contributes to muscle atrophy regulation in mice via the integrin *α7β1* signaling axis [16] and exhibits high expression levels in the *longissimus dorsi* muscle of Polish White pigs [15], its functional network in ruminant myoblasts remains largely unexplored. Our findings suggest that silencing the *CIB2* gene significantly reduces the proliferative activity of goat myoblasts, as evidenced by decreased CCK-8 absorbance values and a reduced proportion of EdU-positive cells. Mechanistically, *CIB2* interference leads to dramatic downregulation of *Cyclin D*, *Cyclin E*, and *CDK4* transcription levels, accompanied by a marked reduction in S-phase cells and G2-phase arrest. These results align closely with the established roles of key cell cycle regulators: the *Cyclin D*–*CDK4*/*6* complex functions as a “molecular switch” for the G1/S phase transition [31], and inhibition of its expression directly impedes cell cycle initiation. Similarly, the absence of *Cyclin E*–*CDK2* disrupts pre-replication complex assembly, thereby obstructing progression through the S phase [32]. It is noteworthy that despite the significant upregulation of the pro-apoptotic gene *Bax* following *CIB2* interference, flow cytometry analysis revealed no significant increase in apoptosis rates. This implies that under short-term intervention, the loss of *CIB2* may predominantly influence cell fate through cell cycle arrest rather than via the apoptotic pathway. This observation aligns with the adaptive regulatory mechanisms of muscle development, wherein cells tend to enter a quiescent state (e.g., re-entering the G0 phase) upon impairment of proliferation signals, rather than initiating apoptosis, thereby preserving regenerative potential [33,34].

Previous studies have demonstrated that the expression of the *CIB2* gene is significantly positively correlated with different developmental stages of goat skeletal muscle and promotes myoblast proliferation by regulating the G1/S phase transition. To further investigate the role of *CIB2* in postnatal muscle regeneration in goats, this study focused on MuSCs, a type of adult stem cell characterized by self-renewal, proliferation, differentiation, and myofiber formation capabilities [35]. Using the goat MuSC model characterized by nuclear *Pax7* expression, we demonstrated that transfection with *CIB2* shRNA significantly decreased cell viability, as evidenced by reduced CCK-8 absorbance. This proliferative defect was further substantiated by EdU incorporation assays, which revealed a 63% reduction in EdU-positive cells. Consistent with these findings, the expression levels of key proliferation markers *PCNA* and *Cyclin E* were markedly downregulated. As a core factor for DNA replication during the S phase [36], the suppression of *PCNA* expression directly supports the molecular mechanism by which *CIB2* regulates MuSC expansion through G1/S-phase control. Notably, this result is functionally complementary to the G2-phase arrest observed in myoblasts upon *CIB2* deficiency.

Further investigation revealed that while silencing *CIB2* inhibits MuSC proliferation, it significantly activates the differentiation program: key myogenic factors such as *MyoD*, *MyoG*, and *MyHC* are upregulated, and myotube formation is accelerated. This phenomenon aligns closely with the cascade regulation network of myogenic factors—during embryonic development, *Pax3*/*Pax7* activation of *MyoD* drives the proliferation of myogenic progenitor cells, followed by *MyoD*-induced expression of *MyoG* and suppression of *Myf5*, initiating differentiation and fusion into myotubes, ultimately maintained by *Mrf4* [37,38]. Importantly, *MyoG*, a critical differentiation factor, promotes MuSC exit from the proliferation state by inhibiting cell cycle-related genes such as *Cyclin E* [39], consistent with the observed downregulation of *Cyclin E* and activation of differentiation following *CIB2* silencing in this study. Additionally, the activation of MuSCs after muscle injury and the upregulation of *Myf5*/*MyoD* further underscore the central role of the dynamic balance of myogenic factors in the proliferation–differentiation transition [40]. Although other regulatory factors, such as *MEF2*, *FGF*, and *IGF*, also contribute to MuSC fate determination [41], this study suggests that *CIB2* may regulate the differentiation process by interfering with the *MyoD*-*MyoG* axis, providing new insights into the mechanism of muscle regeneration.

It is noteworthy that bioinformatic analyses in conjunction with ChIP-qPCR experiments identified a significant binding peak of *CTCF* within the region 54552400–54552700 of the *CIB2* gene. This finding suggests that *CTCF* may regulate the transcriptional activity of *CIB2* by directly binding to its DNA locus, thereby influencing skeletal muscle development. Skeletal muscle regeneration depends on the activation, differentiation, and multinucleated myotube formation of MuSCs. Activated MuSCs must exit the cell cycle, initiate the differentiation program, and ultimately achieve fusion through processes such as cell migration, interactions of membrane surface adhesion molecules, and reorganization of the actin cytoskeleton [42,43,44]. During this process, epigenetic regulators like *CTCF* may precisely control cell proliferation and differentiation by coordinating the dynamic balance of gene expression [45,46]. While prior studies have demonstrated that *CTCF* inhibits *POLD1* transcription by binding to its promoter region, thereby accelerating cellular senescence [47], the regulatory network of *CTCF* in muscle development remains to be elucidated [48]. In this study, interference with the *CTCF* gene significantly upregulated the expression of *CIB2*, indicating that *CTCF* may function as a transcriptional repressor of *CIB2* and play a role in regulating muscle regeneration.

To further elucidate the molecular mechanism underlying *CIB2* regulation of goat MuSC proliferation and differentiation, this study established *CIB2* gene interference and overexpression models in goat myogenic satellite cells and performed RNA-seq transcriptome analysis. KEGG enrichment analysis of DEGs revealed that the *CIB2* regulatory network was significantly enriched in biological processes such as cell cycle regulation, the PI3K-Akt signaling pathway, and ECM–receptor interaction. Notably, the PI3K/AKT/mTOR pathway serves as a core signaling hub for regulating myogenesis, and its activation state dynamically balances the processes of cell proliferation and differentiation. Specifically, AKT promotes the expression of cell cycle proteins such as *Cyclin D*/*E* by phosphorylating downstream targets, thereby driving G1/S phase transition; meanwhile, mTOR coordinates the synthesis of biomacromolecules required for myotube fusion through regulation of ribosome biogenesis and protein translation [49].

Integrins, which are transmembrane receptors composed of α and β subunits, bind to ECM proteins such as fibronectin, collagen, and laminin. This binding activates key intracellular signaling pathways, including focal adhesion kinase (FAK), phosphatidylinositol 3-kinase/protein kinase B (PI3K/Akt), and mitogen-activated protein kinase (MAPK), thereby regulating cell adhesion, migration, proliferation, and differentiation. Conversely, intracellular signals can enhance integrin–ECM interactions by inducing a high-affinity conformational state in integrins [50]. Collagen, as a major constituent of the ECM, plays a critical role in the self-renewal of satellite cells in skeletal muscle. Studies have demonstrated that type VI collagen (*COL6*), an essential component of the satellite cell niche, is crucial for muscle regeneration. Mice deficient in the α1 chain of *COL6* (Col6a1^−/−^) exhibit impaired muscle regeneration and reduced satellite cell self-renewal capacity following injury [51]. Transcriptome sequencing data reveal that *CIB2* knockdown in goat myogenic satellite cells upregulates the expression of collagen genes *COL4* and *COL6*, corroborating these findings. Furthermore, integrin activation triggers focal adhesion kinase (FAK) activation, leading to sequential phosphorylation of downstream pathways such as PI3K/AKT [52,53]. This cascade regulates the expression of cyclins and cyclin-dependent kinases (CDKs), thereby determining the fate of satellite cells [54]. The literature indicates that *GRP94* inhibits the PI3K/AKT/mTOR pathway, thereby inducing myoblast differentiation, whereas *VEGFB* activates the VEGFR1-PI3K/AKT axis to facilitate both myoblast proliferation and differentiation. Additionally, the WDR13 gene enhances the differentiation of bovine skeletal muscle satellite cells by suppressing the PI3K/AKT pathway, underscoring the bidirectional regulation of the PI3K/AKT signaling pathway as a critical molecular switch during myogenesis [55,56].

## 5. Conclusions

**Figure 6:**
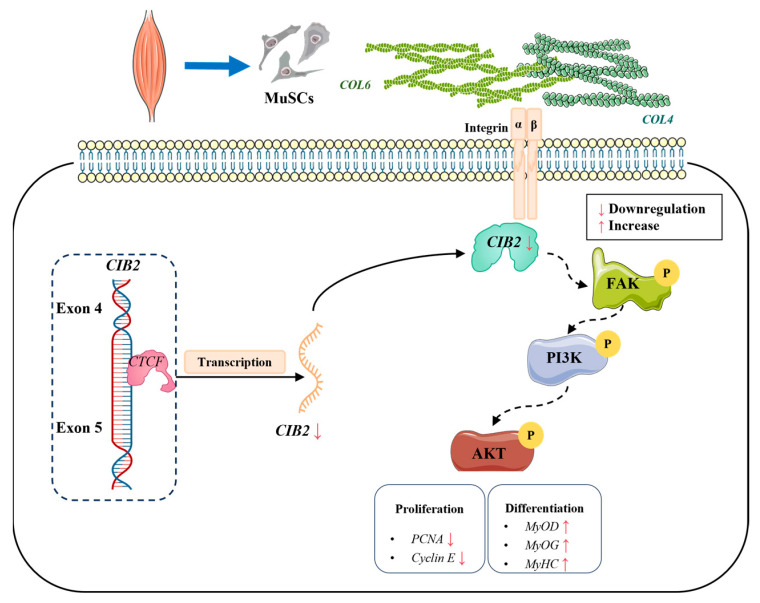
Schematic diagram showing that *CIB2* regulates cell proliferation and differentiation in goat myogenic satellite cells. Transcriptional repression of *CIB2* by *CTCF* modulates integrin α7β1–PI3K/AKT signaling, suppressing proliferation through CDK4/cyclin D/E-dependent S-phase arrest while promoting terminal differentiation via *MyoD*/*MyoG*/*MyHC* activation.

This study demonstrates that CIB2 exerts dual regulatory roles in goat MuSCs, suppressing cell cycle progression via S-phase arrest (CDK4/cyclin D/E-dependent) while promoting terminal differentiation through the *MyoD*/*MyoG*/*MyHC* axis. Critically, we establish the CTCF-CIB2–integrin axis, where the transcriptional repressor *CTCF* negatively regulates *CIB2* expression—revealing its essential function in caprine muscle development (Figure 6). These findings enhance our molecular understanding of genetic traits in ruminants and provide a robust framework for optimizing breeding strategies, such as targeting the CTCF-CIB2 axis, to improve muscle yield and adaptability in sustainable goat production systems.

## Figures and Tables

**Table 1:** Transcription factor prediction results of *CIB2* gene.

Potential Transcription Factors	Cistrome DB (ID)	Peak Intensity	Putative Binding Regions
*CTCF*	34432	4.20	54552400–54552700
*SMAD3*	335	0.60	54554600–54555200
*SPI1*	34422	0.58	54552200–54553100
*SRF*	34430	0.48	54553000–54553600
*YY1*	57269	0.31	54554500–54555100
*TRIM28*	51766	0.30	54554000–54554650
*TEAD4*	74419	0.30	54552500–54553000
*CEBPB*	34428	0.28	54552900–54553600

## Data Availability

The datasets used and analyzed in this study are available from the corresponding author upon reasonable request.

## Supplementary Materials

The following supporting information can be downloaded at https://www.mdpi.com/article/10.3390/cells14151199/s1. Table S1: The sequences of siRNA and shRNA. Table S2: Primer sequences for ChIP-qPCR amplification. Table S3: The sequences of primers for RT-qPCR in this article. Table S4: Statistics of sequencing data output. Figure S1: Analysis of interaction between *CIB2* and integrin α7β1. Figure S2: Transcriptome sequencing analysis of goat myogenic satellite cells after overexpression with *CIB2*.

## Abbreviations

The following abbreviations are used in this manuscript:

CIB2	Calcium- and Integrin-Binding Family Member 2
ECM	Extracellular Matrix
MuSCs	Muscle Satellite Cells
LBW	Low Birth Weight
DEGs	Differentially Expressed Genes
EdU	5-Ethynyl-2′-Deoxyuridine
ChIP-qPCR	Chromatin Immunoprecipitation–Quantitative PCR
RNA-seq	RNA Sequencing
CTCF	CCCTC-Binding Factor (transcriptional repressor)
PI3K/AKT	Phosphoinositide 3-Kinase/Protein Kinase B Pathway
FAK	Focal Adhesion Kinase
CDK4	Cyclin-Dependent Kinase 4
MyHC	Myosin Heavy Chain
