# Supplementary material for: CTCF Represses CIB2 to Balance Proliferation and Differentiation of Goat Myogenic Satellite Cells via Integrin α7β1–PI3K/AKT Axis
Source: Cells. 2025 Aug 5;14(15):1199. doi: 10.3390/cells14151199 (PMC12345746; doi:10.3390/cells14151199)
Supplement: Supplementary file 1 [file cells-14-01199-s001.zip › Figure.pdf]

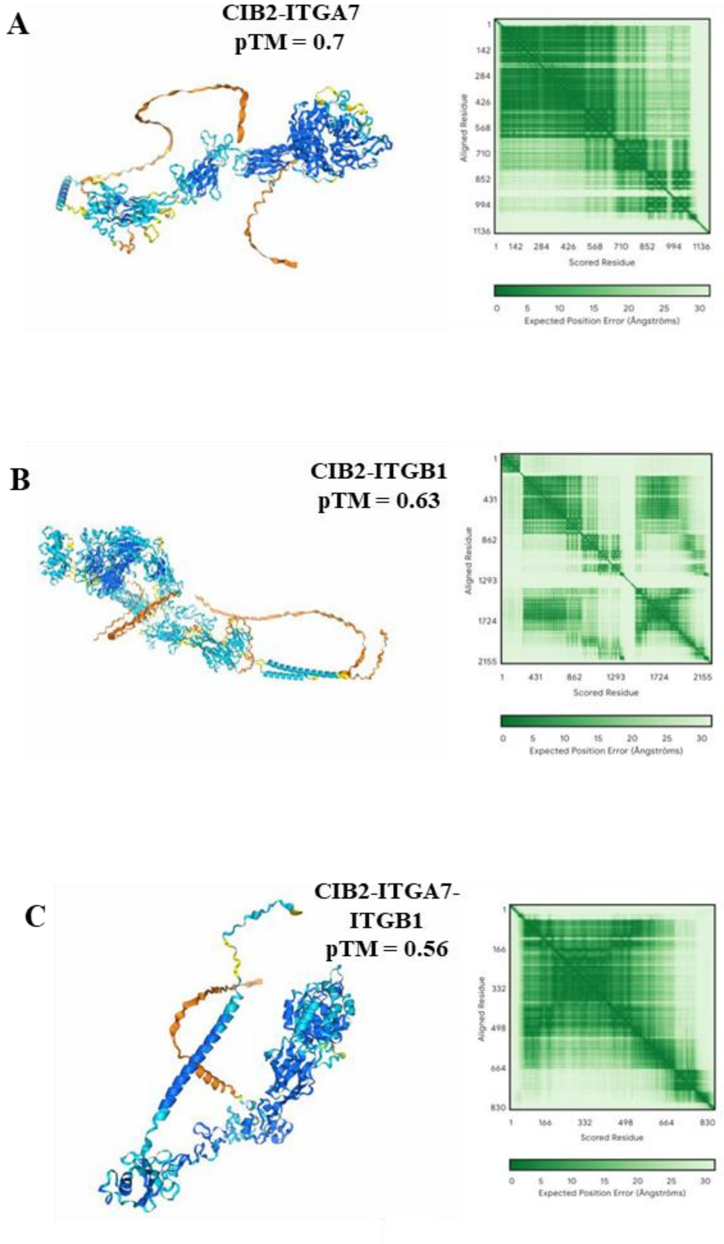

Figure S1. Analysis of interaction between *CIB2* and α7β1. A-C. AlphaFold 3 predicts the binding interaction between CIB2 and integrin α7β1, with the predicted interaction scores (pTM) of CIB2-ITGA7, CIB2-ITGB1, and CIB2-ITGA7-ITGB1 complex shown as indicated.

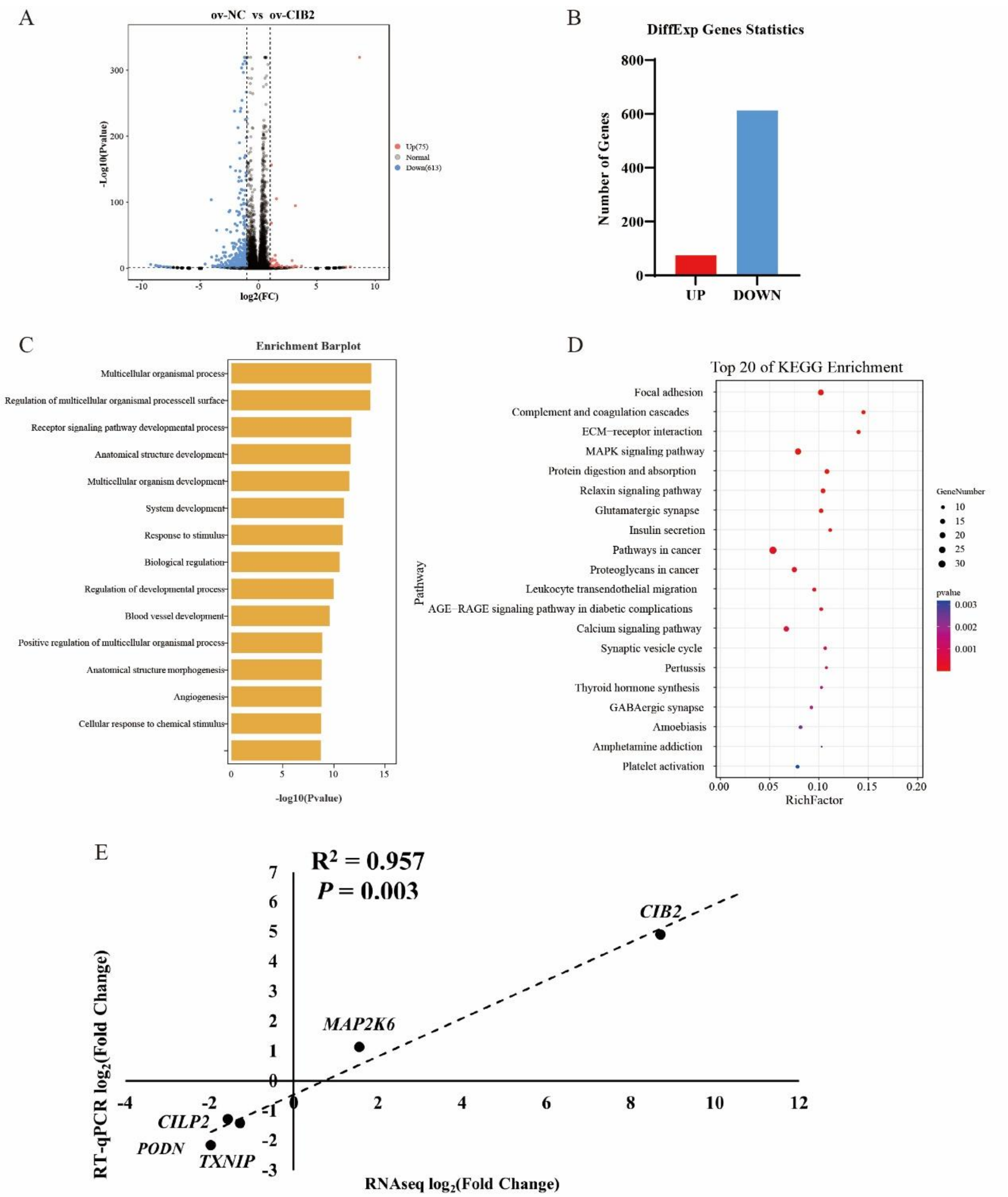

Figure S2. Transcriptome sequencing analysis of goat myogenic satellite cells after overexpression with *CIB2*. A. Volcano map of differentially expressed genes with overexpression *CIB2*. B. The number of differentially expressed genes with overexpression *CIB2*. C. GO BP enrichment analysis following *CIB2* overexpression highlighted the top 15 terms with the lowest *P*-values. D. KEGG enrichment analysis of differentially expressed genes with overexpression *CIB2*. E. Validation of RNA-seq data in the overexpression *CIB2* group by RT-qPCR. The slope of best fit after Pearson correlation was 0.978, with a 95% confidence interval of 0.476 to 0.928.
