# Supplementary material for: CTCF Represses CIB2 to Balance Proliferation and Differentiation of Goat Myogenic Satellite Cells via Integrin α7β1–PI3K/AKT Axis
Source: Cells. 2025 Aug 5;14(15):1199. doi: 10.3390/cells14151199 (PMC12345746; doi:10.3390/cells14151199)
Supplement: Supplementary file 1 [file cells-14-01199-s001.zip › Table S1.pdf]

Table S1 The sequences of siRNA and shRNA

| Gene Name | Cell Type | Sequence（5′-3′） | |
|---|---|---|---|
| shNC | Mouse C2C12 | GGGTGAACTCACGTCAGAATTCAAGAGATTCTGACGTGAGTTCACCC | |
| shCIB2-1 | | CTGCACTTTCTTCAATAAGAAGTTCAAGAGACTTCTTATTGAAGAAAGTGCAG | |
| shCIB2-2 | | CACCTTCAATGACTTTGTGGACTTCAAGAGAGTCCACAAAGTCATTGAAGGTG | |
| shCIB2-3 | | CTGTAAAGAAGACTTAGAGATTTCAAGAGAATCTCTAAGTCTTCTTTACAG | |
| | | Forward（5′-3′） | Reverse (5′-3′) |
| siNC | Mouse C2C12 | UUCUCCGAACGUGUCACGUTT | ACGUGACACGUUCGGAGAATT |
| siCIB2-1 | | GAAUCCCUUCAAAGAGAGGAUTT | AUCCUCUCUUUGAAGGGAUUCTT |
| siCIB2-2 | | CUUUGAGGACAUGAUCGCCAATT | UUGGCGAUCAUGUCCUCAAAGTT |
| siNC | Goat MuSCs | UUCUCCGAACGUGUCACGUTT | ACGUGACACGUUCGGAGAATT |
| siCIB2-1 | | GGAGAAUCCCUUCAAGGAATT | UUCCUUGAAGGGAUUCUCCTT |
| siCIB2-2 | | CCUUCAAGAUCUACGACUUTT | AAGUCGUAGAUCUUGAAGGTT |
| siNC | Mouse C2C12 | UUCUCCGAACGUGUCACGUTT | ACGUGACACGUUCGGAGAATT |
| siCTCF-1 | | CCUUGCAGGUUGUAAAUAUTT | AUAUUUACAACCUGCAAGGTT |
| siCTCF-2 | | GCGGCAUCGUCGUUACAAATT | UUUGUAACGACGAUGCCGCTT |
