# Supplementary material for: CTCF Represses CIB2 to Balance Proliferation and Differentiation of Goat Myogenic Satellite Cells via Integrin α7β1–PI3K/AKT Axis
Source: Cells. 2025 Aug 5;14(15):1199. doi: 10.3390/cells14151199 (PMC12345746; doi:10.3390/cells14151199)
Supplement: Supplementary file 1 [file cells-14-01199-s001.zip › Table S2.pdf]

Table S2 Primer sequences for ChIP-qPCR amplification

| Site Name | Forward (5′-3′) | Reverse (5′-3′) |
|---|---|---|
| Site a | GGGAACAAGCTGTGTGGTAGT | CAGGCCAGAGATAGGGTCATC |
| Site b | GTACTGGGATCTACAGGGCTTG | CGAGCTGGAGGTTCTTCTGTC |
| Site c | CCGAGAGGTGACTTCTCCCT | TCAGTGTCCAGTAGTCAGGCG |
| Site d | GGTCTCACAATGTATCCCAGGTT | CACTTGGGCTTTGGCAGGAT |
| Site e | GTGTATAGTTTGTTTGGTGGTATATTC | CAGATTAAAGGCATCAAAGAGC |
