# Supplementary material for: CTCF Represses CIB2 to Balance Proliferation and Differentiation of Goat Myogenic Satellite Cells via Integrin α7β1–PI3K/AKT Axis
Source: Cells. 2025 Aug 5;14(15):1199. doi: 10.3390/cells14151199 (PMC12345746; doi:10.3390/cells14151199)
Supplement: Supplementary file 1 [file cells-14-01199-s001.zip › Table S3.pdf]

Table S3 The sequences of primers for RT-qPCR

Gene Name	Species	Forward (5′-3′)	Reverse (5′-3′)
Bax	Mouse	TTGCCCTCTTCTACTTTGCTAG	CCATGATGGTTCTGATCAGCTC
Bcl-2		TTGCCCTCTTCTACTTTGCTAG	CCATGATGGTTCTGATCAGCTC
CDK4		CGAGCGTAAGGCTGATGGAT	TCAGGTCCCGGTGAACAATG
Cyclin D		TAGGCCCTCAGCCTCACTC	CCACCCCTGGGATAAAGCAC
Cyclin E		CAGAGCAGCGAGCAGGAGC	GCAGCTGCTTCCACACCACT
GAPDH		TCATCAACGGGAAGCCCATC	TCTCGTGGTTCACACCCATC
CIB2		TCGAAAGGGCGAGAGACGAC	TGAAGAAAGTGCAGTCCTGGTA
Bax	Goat	CCTTGGCTGAGTCGCTGAA	AGCACTCCAGCCACAAAGAT
Bcl-2		CTGTGGATGACCGAGTACCTGAAC	GCCAGACTGAGCAGTGCCTTC
Caspase 3		AGGCAGACTTCTTGTACGCA	TCTGTCGCTACCTTTCGGTT
CDK2		AGCTTTTGGGGTCCCTGTTC	CGGGTCACCATCTCAGCAAA
CDK4		TGTTGTCAGGCTCATGGACG	GGGGTGCCTTGTCCAGATAC
Cyclin E		GCCTGTACTGAACTGGGCAA	GAAGAGGGTGCCGTTGCATA
GAPDH		TTATGACCACTGTCCACGCC	TCAGATCCACAACGGACACG
Ki67		CAATCCAGAAGGGAAAGCTCAAG	GGAACGAACATGACTGGGTGT
MyHC		CAAGGGTCTACGCAAACACGA	AGCTTGCGGAATTTGGAGAGG
MyoD		GCACGTCTAGCAACCCAAAC	AGTCGCCGCTGTAGTGTTC
MyoG		ACCTCACTTCTATGACGGGGA	CTCTTGCACACCTTACACGC
Pax7		CCCTCAGGTTTAGTGAGTTCGATT	AGAGGGAGATCGGGTTCTGAC
PCNA		CCTTGGTGCAGCTAACCCTT	TGCCAAGGTGTCCGCATTAT
CILP2		AGAAATGTCTGCCCCTTCGG	TCCCCGAGGGATATGGTGTGTGTT
TXNIP		GGTGTCTCTCTGCACGAA	CAGAGGAGTAGTGGGGCTCT
PODN		ACGGCTCGACTTGAATGGAA	GGGATGATCTGACCTGCTGC
HSPA6		CGTGAGGCTGAGCAGTACAA	GTCCACAAACCCAGCCCTTA
MAP2K6		CAGCCTTCCCTAACGTTGC	GTTTCGCTTCTTGCCTTTCG
IGFBP2		CGAGCAGGTTGCAGACAATG	GAGGTTGTACAGGCCATGCT
CIB2		GGACATCCTCAAGCTCCACG	CCCCTCACACCCTCAGAGA
