# Supplementary material for: CTCF Represses CIB2 to Balance Proliferation and Differentiation of Goat Myogenic Satellite Cells via Integrin α7β1–PI3K/AKT Axis
Source: Cells. 2025 Aug 5;14(15):1199. doi: 10.3390/cells14151199 (PMC12345746; doi:10.3390/cells14151199)
Supplement: Supplementary file 1 [file cells-14-01199-s001.zip › Table S4.pdf]

Table S4 Statistics of sequencing data output.

#SampleID	RawReads	CleanReads	GC(%)	CleanRatio	Q20(%)	Q30(%)
pcDNA3_1_CIB2_1	38630364	38347140	48.68%	99.27%	96.04%	86.95%
pcDNA3_1_CIB2_2	42453126	42145122	48.51%	99.27%	96.05%	86.97%
pcDNA3_1_CIB2_3	44437824	44110146	48.60%	99.26%	96.87%	89.10%
pcDNA3_1_NC1	42102356	41799346	48.50%	99.28%	96.02%	86.88%
pcDNA3_1_NC2	43613772	43300212	48.51%	99.28%	95.87%	86.35%
pcDNA3_1_NC3	39829156	39538792	48.44%	99.27%	96.79%	88.81%
siCIB2_1	40102546	39808322	48.45%	99.27%	96.58%	88.32%
siCIB2_2	95771174	95693778	47.59%	99.92%	97.50%	92.16%
siCIB2_3	96925870	96848206	47.85%	99.92%	97.35%	91.68%
siNC_1	187138150	186995858	47.07%	99.92%	97.32%	91.58%
siNC_2	103443372	103362210	47.42%	99.92%	97.34%	91.64%
siNC_3	112241022	112150194	47.52%	99.92%	97.29%	91.48%

Annotation:
The Quality Score is an overall mapping of the probability of base calling errors. The base quality value Q formula is: $Q\text{-score} = -10*\log_{10}P$, where P is the probability of base calling error. The following table shows the correspondence between the base quality value and the probability of a base call error:

Base quality score	Probability of base calling errors	Base calling accuracy
10	10%	90%
20	1%	99%
30	0.1%	99.9%
40	0.01%	99.99%
